# Sample size calculations for model validation in linear regression analysis

**DOI:** 10.1186/s12874-019-0697-9

**Published:** 2019-03-12

**Authors:** Show-Li Jan, Gwowen Shieh

**Affiliations:** 1 Department of Applied Mathematics, Chung Yuan Christian University, Taoyuan, Taiwan 32023 Republic of China; 2 Department of Management Science, National Chiao Tung University, Hsinchu, Taiwan 30010 Republic of China

**Keywords:** Linear regression, Model validation, Power, Sample size, Stochastic predictor

## Abstract

**Background:**

Linear regression analysis is a widely used statistical technique in practical applications. For planning and appraising validation studies of simple linear regression, an approximate sample size formula has been proposed for the joint test of intercept and slope coefficients.

**Methods:**

The purpose of this article is to reveal the potential drawback of the existing approximation and to provide an alternative and exact solution of power and sample size calculations for model validation in linear regression analysis.

**Results:**

A fetal weight example is included to illustrate the underlying discrepancy between the exact and approximate methods. Moreover, extensive numerical assessments were conducted to examine the relative performance of the two distinct procedures.

**Conclusions:**

The results show that the exact approach has a distinct advantage over the current method with greater accuracy and high robustness.

**Electronic supplementary material:**

The online version of this article (10.1186/s12874-019-0697-9) contains supplementary material, which is available to authorized users.

## Background

Regression analysis is the most commonly applied statistical method of all scientific fields. The extensive utility incurs continuous investigations to give various interpretations, extensions, and computing algorithms for the development and formulation of empirical models. General guidelines and fundamental principles on regression analysis have been well documented in the standard texts of Cohen et al. [1], Kutner et al. [2], and Montgomery, Peck, and Vining [3], among others. Among the methodological issues and statistical implications of regression analysis, model adequacy and validity represent two vital aspects for justifying the usefulness of the underlying regression model. In the process of model selection, residual analysis and diagnostic checking are employed to identify influential observations, leverage, outliers, multicollinearity, and other lack of fit problems. Alternatively, model validation refers to the plausibility and generalizability of the regression function in terms of the stability and suitability of the regression coefficients.

In particular, it is emphasized in Kutner et al. ([2], Section 9.6), Montgomery, Peck, and Vining ([3], Section 11.2), and Snee [4] that there are three approaches to assessing the validity of regression models: (1) comparison of model predictions and coefficients with physical theory, prior experience, theoretical models, and other simulation results; (2) collection of new data to check model predictions; and (3) data splitting in which reservation of a portion of the available data is used to obtain an independent measure of the model prediction accuracy. Essentially, the fundamental utilities between model selection and model validation should be properly recognized and distinguished because a refined model that fits the data does not necessarily guarantee prediction accuracy. Further details and related issues can be found in the importance texts of Kutner et al. [2] and Montgomery, Peck, and Vining [3] and the references therein.

The present article focuses on the validation process of linear regression analysis for comparison with postulated or acclaimed models. In linear regression, the focus is often concerned with the existence and magnitude of the slope coefficients. However, the quality of estimation and prediction in associating the response variable with the predictor variables is determined by the closely intertwined intercept and slope coefficients. It is of practical importance to conduct a joint test of intercept and slope coefficients in order to verify the compatibility with established or theoretical formulations. For example, Maddahi et al. [5] compared left ventricular myocardial weights of dogs by nuclear magnetic resonance imaging with actual measurements for different methods using simple linear regression analysis. The results were tested, both individually and simultaneously, whether the intercept was different from zero and the slope was different form unity. Also, Rose and McCallum [6] proposed a simple regression formula for estimating the logarithm of feta weight with the sum of the ultrasound measurements of biparietal diameter, mean abdominal diameter, and femur length. Note that the birth weights differ among ethic groups, cohort characteristics, and time periods. Thus, it is of considerable interest for related research to validate or compare the magnitudes of intercept and slope coefficients in their formulation.

The importance and implications of statistical power analysis in research studies are well addressed in Cohen [7], Kramer and Blasey [8], Murphy, Myros, and Wolach [9], and Ryan [10], among others. In the context of multiple regression and correlation, the distinct notions of fixed and random regression settings were emphasized and explicated in power and sample size calculations by Gatsonis and Sampson [11], Mendoza and Stafford [12], Sampson [13], and Shieh [14–16]. On the other hand, Kelley [17], Krishnamoorthy and Xia [18], and Shieh [19] discussed sample size determinations for constructing precise confidence intervals of strength of association. It is noteworthy that analysis of covariance (ANCOVA) models involving both categorical and continuous predictors incur different hypothesis testing procedures. Accordingly, they require unique power procedures as discussed in Shieh [20] and Tang [21], among others.

For the purposes of planning research designs and validating model formulation, a sample size procedure was presented in Colosimo et al. [22]. The presented formula has a computationally appealing expression and maintains reasonable accuracy in their simulation study. However, the particular method involves a convenient substitution of fixed mean parameter for random predictor variables. Their illustrations were not detailed enough to address the extent and impact of such simplification in sample size computations. Consequently, the adequacy of the sample size procedure described in Colosimo et al. [22] requires further clarification and no research to date has examined its properties under different situations.

The statistical inferences for the regression coefficients are based on the conditional distribution of the continuous predictors. However, unlike the fixed factor configurations and treatment levels in analysis of variance (ANOVA) and other experimental designs, the continuous measurements of the predictor variables in regression studies are typically available only after the data has been collected. For advance planning research design, the distribution and power functions of the test procedure need to be appraised over possible values of the predictors. Thus, it is important to recognize the stochastic nature of the predictor variables. The fundamental differences between fixed and random models have been explicated in Binkley and Abbot [23], Cramer and Appelbaum [24], Sampson [13], and Shaffer [25]. Despite the complexity associated with the unconditional properties of the test procedure, the inferential procedures are the same under both fixed and random formulations. Hence, the usual rejection rule and critical value remain unchanged. The distinction between the two modeling approaches becomes critical for power analysis and sample size planning.

The joint test of intercept and slope coefficients in linear regression is more involved than the individual tests of intercept or slope parameters. A general linear hypothesis setting is required to perform the simultaneous test of both intercept and slope coefficients as shown in Rencher and Schaalje ([26], Section 8.4.2). However, it is essential to emphasize that they did not address the corresponding power and sample size issues. In view of the limited results in current literature, this article aims to present power and sample size procedure for the joint test of intercept and slope coefficients with specific recognition of the stochastic features of predictor variables. First, exact power function and sample size procedure for detecting intercept and slope differences of simple linear regression are derived under random modeling framework assuming predictor variables have independent and identical normal distribution. Then, the technical presentation is extended to the general context of multiple linear regression. Then, a numerical example of model validation is employed to demonstrate the essential discrepancy between the exact and approximate methods. The accuracy and robustness of the contending methods are appraised through simulation studies under a wide range of model configurations with normal and non-normal predictors.

## Methods

### Simple linear regression

Consider the simple linear regression model for associating the response variable *Y* with the predictor variable *X*:

1 $$ {Y}_i={\upbeta}_I+{X}_i{\upbeta}_S+{\upvarepsilon}_{i,} $$

where *Y*_*i*_ is the observed value of the response variable *Y*; *X*_*i*_ is the recorded value of the continuous predictor *X*; β_*I*_ and β_*S*_ are unknown intercept and slope parameters; and ε_*i*_ are *iid N*(0, σ^2^) random errors for *i* = 1, …, *N*. To examine the existence and magnitude of the intercept and slope coefficients {β_*I*_, β_*S*_}, the statistical inferences are based on the least squares estimators ${\widehat{\upbeta}}_I$ and ${\widehat{\upbeta}}_S$, where ${\widehat{\upbeta}}_I$ = $\overline{Y}$ – $\overline{X}{\widehat{\upbeta}}_S$, ${\widehat{\upbeta}}_S$ = *SSXY*/*SSX*, $\overline{Y}$ = $\sum \limits_{i=1}^N$*Y*_*i*_/*N*, $\overline{X}$ = $\sum \limits_{i=1}^N$*X*_*i*_/*N*, *SSXY* = $\sum \limits_{i=1}^N$(*X*_*i*_ – $\overline{X}$)(*Y*_*i*_ – $\overline{Y}$), and *SSX* = $\sum \limits_{i=1}^N$(*X*_*i*_ – $\overline{X}$)^2^. It follows from the standard results in Rencher and Schaalje ([26], Section 7.6.3) that the estimators {${\widehat{\upbeta}}_I$, ${\widehat{\upbeta}}_S$} have the bivariate normal distribution

2 $$ \widehat{\boldsymbol{\upbeta}}\sim {N}_2\left(\boldsymbol{\upbeta}, {\upsigma}^2{\mathrm{W}}_X\right), $$

where

$$ \widehat{\boldsymbol{\upbeta}}=\left[\begin{array}{c}{\widehat{\upbeta}}_I\\ {}{\widehat{\upbeta}}_S\end{array}\right],\kern0.5em \boldsymbol{\upbeta} =\left[\begin{array}{c}{\upbeta}_I\\ {}{\upbeta}_S\end{array}\right],\kern0.5em {\mathbf{W}}_X=\left[\begin{array}{cc}{W}_{X11}& {W}_{X12}\\ {}{W}_{X21}& {W}_{X22}\end{array}\right], $$

*W*_*X*11_ = 1/*N* + ${\overline{X}}^2$/*SSX*, *W*_*X*12_ = *W*_*X*21_ = −$\overline{X}$/*SSX*, and *W*_*X*22_ = 1/*SSX*. The subscript *X* of **W**_*X*_ emphasizes the elements {*W*_*X*11_, *W*_*X*12_, *W*_*X*21_, *W*_*X*22_} of the variance and covariance matrix are functions of the predictor variables. Also, ${\widehat{\upsigma}}^2$ = *SSE*/ν is the usual unbiased estimator of σ^2^, where *SSE* = *SSY* – *SSXY*^2^/*SSX* is the error sum of squares, *SSY* = $\sum \limits_{i=1}^N$(*Y*_*i*_ – $\overline{Y}$)^2^, and ν = *N* – 2. Note that the least squares estimators ${\widehat{\upbeta}}_I$ and ${\widehat{\upbeta}}_S$ are independent of ${\widehat{\upsigma}}^2$.

A joint test of the intercept and slope coefficients can be conducted with the hypothesis

3 $$ {\mathrm{H}}_0:\kern1em \left[\begin{array}{c}{\upbeta}_I\\ {}{\upbeta}_S\end{array}\right]=\left[\begin{array}{c}{\upbeta}_{I0}\\ {}{\upbeta}_{S0}\end{array}\right]\;\mathrm{versus}\ {\mathrm{H}}_1:\kern0.5em \left[\begin{array}{c}{\upbeta}_I\\ {}{\upbeta}_S\end{array}\right]\ne \left[\begin{array}{c}{\upbeta}_{I0}\\ {}{\upbeta}_{S0}\end{array}\right]. $$

Following the model assumption in Eq. 1, the likelihood ratio statistic for the joint test of intercept and slope is

4 $$ {F}_J=\frac{\left({\widehat{\boldsymbol{\upbeta}}}_D^{\mathrm{T}}{\mathbf{W}}_X^{\hbox{-} 1}{\widehat{\boldsymbol{\upbeta}}}_D\right)/2}{{\widehat{\sigma}}^2}, $$

where ${\widehat{\boldsymbol{\upbeta}}}_D$ = [${\widehat{\upbeta}}_{ID}$, ${\widehat{\upbeta}}_{SD}$]^T^, ${\widehat{\upbeta}}_{ID}$ = ${\widehat{\upbeta}}_I$ – β_*I*0_, and ${\widehat{\upbeta}}_{SD}$ = ${\widehat{\upbeta}}_S$ – β_*S*0_. Under the null hypothesis, it can be shown that

5 $$ {F}_J\sim F\left(2,\upnu \right), $$

where *F*(2, ν) is an *F* distribution with 2 and ν degrees of freedom. Hence, *H*_0_ is rejected at the significance level α if

6 $$ {F}_J>{F}_{2,\upnu, \upalpha}, $$

where *F*_2, ν, α_ is the upper (100·α)th percentile of the *F*(2, ν) distribution. In general, the joint test statistic *F*_*J*_ has the nonnull distribution for the given values of $\overline{X}$ and *SSX*:

7 $$ {F}_J\left|\left[\overline{X}, SSX\right]\sim F\left(2,\upnu, {\Delta}_J\right)\right. $$

where

8 $$ {\Delta}_J=\kern0.5em \frac{\left\{N{\left({\upbeta}_{ID}+\overline{X}{\upbeta}_{SD}\right)}^2+{\upbeta}_{SD}^2 SSX\right\}}{\upsigma^2}. $$

Hence, the noncentral *F* distribution *F*(*c*, ν, Δ_*J*_) is a function of the predictor values {*X*_*i*_, *i* = 1, …, *N*} only through the summary statistics $\overline{X}$ and *SSX*.

The joint test of the intercept and slope coefficients given in Eq. 3 can be viewed as a special case of the general linear hypothesis considered in Rencher and Schaalje ([26], Section 8.4.2). However, two important aspects of this study should be pointed out. First, unlike the current consideration, the associated *F* test and related statistical properties in Rencher and Schaalje [26] are presented under the standard settings with fixed predictor values. Second, they did not address the power and sample size issues under random modeling formulations. Accordingly, their fundamental results are extended here to accommodate the predictor features in power and sample size calculations for the validation of simple linear regression models.

The statistical inferences about the regression coefficients are based on the conditional distribution of the continuous variables {*X*_*i*_, *i* = 1, …, *N*}. Therefore, the resulting analysis would be specific to the observed values of the predictors. It is clear that, before conducting a research study, the actual values of predictors are not available beforehand just as the major responses. In view of the stochastic nature of the summary statistics $\overline{X}$ and *SSX*, it is essential to recognize and assess the distribution of the test statistic over possible values of the predictors. To demonstrate the impact of the predictor features on power and sample size calculations, the normality setting is commonly employed to provide a convenient basis for analytical derivation and empirical examination of random predictors as in Gatsonis and Sampson [11], Sampson [13], and Shieh [14]. However, it is important to note that the power and sample size calculations of Gatsonis and Sampson [11], Sampson [13], and Shieh [14, 15] for detecting slope coefficients in multiple regression analysis are not applicable for assessing differences in intercept and slope coefficients considered here.

Specifically, the continuous predictor variables {*X*_*i*_, *i* = 1, ..., *N*} are assumed to have independent and identical normal distribution *N*(μ_X_, ${\upsigma}_X^2$). With the normal assumption, it can be readily established that $\overline{X}$ ~ *N*(μ_*X*_, ${\upsigma}_X^2$/*N*) and *K* = *SSX*/${\upsigma}_X^2$ ~ χ^2^(κ) where κ = *N* – 1. Thus, the noncentrality Δ_*J*_ in Eq. 8 can be expressed as

9 $$ {\Delta}_J=\kern0.5em \frac{\left\{N{\left(a+ bZ\right)}^2+ dK\right\}}{\upsigma^2}, $$

where *a* = β_*ID*_ + μ_X_β_SD_, *b* = (*d*/*N*)^1/2^, *d* = ${\upbeta}_{SD}^2{\upsigma}_X^2$, and *Z* = ($\overline{X}$ – μ_*X*_)/(${\upsigma}_X^2$/*N*)^1/2^ ~ *N*(0, 1). As a consequence, the *F*_*J*_ statistic has the two-stage distribution

10 $$ {F}_J\mid \left[K,\kern0.5em Z\right]\kern1em \sim \kern0.5em F\left(2,\kern0.5em \upnu, \kern0.5em {\Delta}_J\right),\kern0.5em K\sim {\upchi}^2\left(\upkappa \right),\mathrm{and}\kern0.5em Z\sim N\left(0,1\right). $$

Note that the two random variables *K* and *Z* are independent. Moreover, the corresponding power function for the simultaneous test can be formulated as

11 $$ {\Psi}_J={E}_K{E}_Z\left[P\left\{F\left(2,\upnu, {\Delta}_J\right)>{F}_{2,v,\alpha}\right\}\right], $$

where the expectations *E*_*K*_ and *E*_*Z*_ are taken with respect to the distributions of *K* and *Z*, respectively.

Alternatively, Colosimo et al. ([22], Section 3.2) described a simple and naive method to obtain an unconditional distribution of *F*_*J*_. They substituted the sample values of the predictor variables in the noncentrality Δ_*J*_ with the corresponding expected value *E*[*X*_*i*_] = μ_X_ for *i* = 1, ..., *N*. Thus, the distribution of *F*_*J*_ is approximated by a noncentral *F* distribution:

12 $$ {F}_J\sim F\left(2,\ \upnu,\ {\Delta}_C\right), $$

where Δ_*C*_ = (*Na*^2^)/σ^2^. The suggested power function of Colosimo et al. [22] for the joint test of intercept and slope coefficients is

13 $$ {\Psi}_C=P\left\{F\left(2,\upnu, {\Delta}_C\right)>{F}_{2,v,\alpha}\right\}. $$

It is vital to note that the approximate power function Ψ_*C*_ only involves a noncentral *F* distribution, whereas the normal predictor distributions lead to the exact and more complex power formula Ψ_*J*_ that consists of a joint chi-square and normal mixture of noncentral *F* distributions. Evidently, the power function Ψ_*C*_ is relatively simpler to compute than the exact formula Ψ_*J*_. But the approximate nature of Ψ_*C*_ does not involve all of the predictor features in power computations.

It follows from large sample theory that *Z* and *K*/*N* converge to 0 and 1, respectively. Hence, the sample-size-adjusted noncentrality quantity Δ_*J*_/*N* approaches ${\Delta}_J^{\ast }$ as the sample size *N* increases to infinity, where

14 $$ {\Delta}_J^{\ast }=\kern0.5em \frac{{\left({\upbeta}_{ID}+{\upmu}_X{\upbeta}_{SD}\right)}^2+{\upbeta}_{SD}^2{\upsigma}_X^2}{\upsigma^2}. $$

Hence, ${\Delta}_J^{\ast }$ provides a convenient measurement of effect size for the joint appraisal of intercept and slope coefficients. It can be immediately seen from the noncentrality term of the approximate power function Ψ_*C*_ that ${\Delta}_C^{\ast }$ = Δ_*C*_/*N* = (β_*ID*_ + μ_*X*_β_*SD*_)^2^/σ^2^ < ${\Delta}_J^{\ast }$ with the exceptions that β_*SD*_ = 0 and/or ${\upsigma}_X^2$ = 0. Consequently, the estimated power Ψ_*C*_ is generally less than that of Ψ_*J*_ even for large sample sizes when all other configurations remain constant. It is shown later that while the computation is more involved for the complex power function Ψ_*J*_, the exact approach has a clear advantage over the approximate procedure in accurate power calculations. For advance planning of a research design, the presented power formulas can be employed to calculate the sample size *N* needed to attain the specified power 1 – β for the chosen significance level α, null values {β_*I*0_, β_*S*0_}, coefficient parameters {β_*I*_, β_*S*_}, variance component σ^2^, and predictor mean and variance {μ_X_, ${\upsigma}_X^2$}. It usually involves an incremental search with a small initial value to find the optimal solution for achieving the desired power performance.

### Multiple linear regression

The power and sample size calculations for the general scenario of multiple linear regression with more than one predictor are discussed next. Consider the multiple linear regression model with response variable *Y*_*i*_ and *p* predictor variables (*X*_*i*1_, ..., *X*_*ip*_) for *i* = 1, ..., *N*:

15 $$ \mathbf{Y}=\mathbf{X}\boldsymbol{\upbeta } +\boldsymbol{\upvarepsilon}, $$

where **Y** = (*Y*_1_, ..., *Y*_*N*_)^T^ is an *N* × 1 vector with *Y*_*i*_ being the observed measurement of the *i*th subject; **X** = (**1**_*N*_, **X**_*S*_) with **1**_*N*_ is the *N* × 1 vector of all 1’s, **X**_*S*_ = (**X**_*S*1_, ..., **X**_*SN*_)^T^ is an *N* × *p* matrix, **X**_*Si*_ = (*X*_*i*1_, ..., *X*_*ip*_)^T^, *X*_*i*1_, ..., *X*_*ip*_ are the observed values of the *p* predictor variables of the *i*th subject; **β** = (β_*I*_, ${\boldsymbol{\upbeta}}_S^{\mathrm{T}}$)^T^ is a (*p* + 1) × 1 vector with **β**_*S*_ = (β_1_, ..., β_*p*_)^T^ and β_*I*_, β_1_, ..., β_*p*_ are unknown coefficient parameters; and **ε** = (ε_1_, ..., ε_*N*_)^T^ is an *N* × 1 vector with ε_*i*_ are *iid N*(0, σ^2^) random variables.

For the joint test of intercept and slope coefficients in terms of

16 $$ {\mathrm{H}}_0:\boldsymbol{\upbeta} =\boldsymbol{\uptheta}\ \mathrm{versus}\ {\mathrm{H}}_1:\boldsymbol{\upbeta} \ne \boldsymbol{\uptheta}, $$

it can be shown from Rencher and Schaalje ([6], Section 8.4.2) that the test statistic is

17 $$ {F}_{MJ}=\frac{\left\{{\left(\widehat{\boldsymbol{\upbeta}}-\boldsymbol{\uptheta} \right)}^{\mathrm{T}}\left({\mathbf{X}}^{\mathrm{T}}\mathbf{X}\right)\left(\widehat{\boldsymbol{\upbeta}}-\boldsymbol{\uptheta} \right)\right\}/\left(p+1\right)}{{\widehat{\upsigma}}^2}, $$

where ${\widehat{\upsigma}}^2$ = *SSE*/ν is the usual unbiased estimator of σ^2^. Under the null hypothesis, *F*_*MJ*_ has an *F* distribution with *p* + 1 and ν degrees of freedom

18 $$ {F}_{MJ}\sim F\left(p+1,v\right) $$

The joint test can be conducted by reject *H*_0_ at the significance level α if *F*_*MJ*_ > *F*_(*p* + 1), ν, α_. In general, *F*_*MJ*_ has the nonnull distribution for the given values of **X**_*S*_:

19 $$ {F}_{MJ}\sim F\left(p+1,\ \upnu,\ {\Delta}_{MJ}\right), $$

where *F*(*p* + 1, ν, Δ_*MJ*_) is a noncentral *F* distribution with *p* + 1 and ν degrees of freedom and noncentrality parameter Δ_*MJ*_ with

20 $$ {\Delta}_{MJ}=\frac{\left\{{\left(\boldsymbol{\upbeta} -\boldsymbol{\uptheta} \right)}^{\mathrm{T}}\left({\mathbf{X}}^{\mathrm{T}}\mathbf{X}\right)\left(\boldsymbol{\upbeta} -\boldsymbol{\uptheta} \right)\right\}}{\upsigma^2}. $$

It is essential to emphasize that the inferences in Rencher and Schaalje [26] are concerned mainly with the slope coefficients **β**_*S*_. As noted in the context of simple linear regression, the fundamental results concerning fixed predictor values are extended here to power and sample size calculations for the validation of linear regression models under random predictor settings.

In view of the random nature of the predictor variables, the continuous predictor variables {**X**_*Si*_, *i* = 1, ..., *N*} are assumed to have independent multinormal distributions *N*_*p*_(**μ**_*X*_, **Σ**_*X*_). With the multinormal assumptions, it can be readily established that ${\overline{\mathbf{X}}}_S$ = $\sum \limits_{i=1}^N$**X**_*Si*_/*N* ~ *N*_*p*_(**μ**_*X*_, **Σ**_*X*_/*N*) and **A** = $\sum \limits_{i=1}^N$(**X**_*Si*_ – ${\overline{\mathbf{X}}}_S$)(**X**_*Si*_ – ${\overline{\mathbf{X}}}_S$)^T^ ~ *W*_*p*_(κ, **Σ**_*X*_), where *W*_*p*_(κ, **Σ**_*X*_) is a Wishart distribution with κ degrees of freedom and covariance matrix **Σ**_*X*_, and κ = *N* – 1. Thus, the noncentrality Δ_*MJ*_ can be rewritten as

21 $$ {\Delta}_{MJ}=\kern0.5em \frac{\left\{N{\left({\upbeta}_{ID}+{\boldsymbol{\upbeta}}_{SD}^{\mathrm{T}}{\overline{\mathbf{X}}}_S\right)}^2+{\boldsymbol{\upbeta}}_{SD}^{\mathrm{T}}{\mathbf{A}\boldsymbol{\upbeta}}_{SD}\right\}}{\upsigma^2}, $$

where β_*ID*_ = β_*I*_ – θ_*I*_ and **β**_*SD*_ = **β**_*S*_ – **θ**_*S*_. Using the prescribed distributions of ${\overline{\mathbf{X}}}_S$ and **A**, it can be shown that β_*ID*_ + ${\boldsymbol{\upbeta}}_{SD}^{\mathrm{T}}{\overline{\mathbf{X}}}_S$ = *a* + *bZ* ~ *N*(*a*, *b*^2^), *Z* ~ *N*(0, 1), and *K* = ${\boldsymbol{\upbeta}}_{SD}^{\mathrm{T}}{\mathbf{A}\boldsymbol{\upbeta}}_{SD}$/*d* ~ χ^2^(κ), where *a* = β_*ID*_ + ${\boldsymbol{\upbeta}}_{SD}^{\mathrm{T}}$**μ**_*X*_, *b* = (*d*/*N*)^1/2^, and *d* = ${\boldsymbol{\upbeta}}_{SD}^{\mathrm{T}}{\boldsymbol{\Sigma}}_X{\boldsymbol{\upbeta}}_{SD}$. Note that the two random variables *K* and *Z* are independent. It is conceptually simple and computationally convenient to subsume the stochastic features of ${\overline{\mathbf{X}}}_S$ and **A** in terms of *Z* and *K*. Accordingly, the noncentrality quantity Δ_*J*_ is formulated as

22 $$ {\Delta}_{MJ}=\frac{\left\{N{\left(a+ bZ\right)}^2+ dK\right\}}{\upsigma^2}. $$

Thus, under the multinormal predictor assumptions, the *F*_*MJ*_ statistic has the two-stage distribution

23 $$ {F}_{MJ}\mid \left[K,Z\right]\sim F\left(p+1,\kern0.5em \upnu, \kern0.5em {\Delta}_{MJ}\right),K\sim {\upchi}^2\left(\upkappa \right),\mathrm{and}\kern0.5em Z\sim N\left(0,1\right). $$

The corresponding power function for the simultaneous test can be termed as

24 $$ {\Psi}_{MJ}={E}_K{E}_Z\left[P\left\{F\left(p+1,\kern0.5em \upnu, \kern0.5em {\Delta}_{MJ}\right)>{F}_{\left(p+1\right),\upnu, \upalpha}\right\}\right], $$

where the expectations *E*_*K*_ and *E*_*Z*_ are taken with respect to the distribution of *K* and *Z*, respectively. Evidently, when *p* = 1, the test statistic *F*_*MJ*_ and power function Ψ_*MJ*_ reduce to the simplified formulas of *F*_*M*_ and Ψ_*J*_ given in Eqs. 4 and 11, respectively.

## Results

### An illustration

To demonstrate the prescribed power and sample size procedures, the simplified formula for estimating fetal weight in Rose and McCallum [6] is used as a benchmark for validation. Although there are several different methods for estimating the fetal weight, it was demonstrated in Anderson et al. [27] that the simple linear regression formula of Rose and McCallum [6] compares favorably with other techniques. Based on the ultrasound examinations conducted in the Stanford University Hospital labor and delivery suite between January 1981 and March 1984, they presented a useful formula for predicting the natural logarithm of birth weight with the sum of head, abdomen, and limb ultrasound measurements as given by the equation: *ln*(BW) = 4.198 + 0.143·*X*, where *X* = biparietal diameter + mean abdominal diameter + femur length (in centimeters). The average birth weight of their study population was 2275 g with a range of 490–5300 g. The detailed comparisons and related discussions of viable equations for estimating fetal weight can be found in Anderson et al. [27] and the references therein.

Conceivably, there are underlying differences in fetal weight between different ethnic origins, cohort groups, and time periods. To validate the simple formula for a target population, it requires a detailed scheme to determine the necessary sample size so that the conducted study has a decent assurance in detecting the potential discrepancy. For illustration, the intercept and slope coefficients are set as β_*I*_ = 4.1 and β_*S*_ = 0.15, respectively. The error component is selected to be σ^2^ = 0.095. The characteristics of the ultrasound measurements are represented by the mean μ_*X*_ = 24.2 and variance ${\upsigma}_X^2$ = 6. Note that these configurations assure that the expected fetal weight of the designated population *E*[BW] = *E*[*exp*(4.1 + 0.15·*X* + ε)] = 2275.52 coincides the average magnitude of birth weighs reported in Rose and McCallum [6]. To test the hypothesis of H_0_: (β_*I*_, β_*S*_) = (4.198, 0.143) versus H_1_: (β_*I*_, β_*S*_) ≠ (4.198, 0.143) with the significance level α = 0.05, numerical computations showed that the sample sizes of *N*_*E*_ = 173 and 227 are required for the exact approach to attain the target power of 0.8 and 0.9, respectively. Because of the sample sizes need to be integer values in practice, the attained power is slightly greater than the nominal power level. In these two cases, the achieved powers of the two sample sizes are Ψ_*J*_ = 0.8001 and 0.9010, respectively. These results were computed with the supplementary algorithms presented in Additional files 1 and 2. For ease of application, the prescribed configurations are incorporated in the user specification sections of the SAS/IML programs.

On the other hand, the matching sample sizes computed with the approximate method of Colosimo et al. [22] are *N*_*C*_ = 183 and 239 with the attained powers of Ψ_*C*_ = 0.8010 and 0.9002, respectively. Therefore, the simple method of Colosimo et al. [22] clearly requires 183–173 = 10 and 239–227 = 12 more babies than the exact formula to satisfy the nominal power performance. Actually, the exact power function gives the values Ψ_*J*_ = 0.8236 and 0.9161 with the sample sizes 183 and 239, respectively. Hence, the resulting power differences between the two magnitudes of sample size are 0.8236–0.8001 = 0.0235 and 0.9161–0.9010 = 0.0151. To enhance the illustration, the computed sample size, estimated power, and difference for the exact and approximate procedures are summarized in Table 1. The sample size and power calculations show that the approximate power function Ψ_*C*_ tends to underestimate powers because the simplification of noncentrality parameter in the noncentral *F* distribution. Correspondingly, the approximate method of Colosimo et al. [22] often overestimates the required sample sizes for validation analysis. It is essential to note that adopting a small sample size will cause a study that has insufficient power to demonstrate model difference. In this case of Colosimo et al. [22], their method may lead to an over-sized study that wastes time, money, and other resources. More importantly, the hypothesis tests of validation studies are over-rejected and yield erroneous conclusions. It is of both practical usefulness and theoretical concern to further assess the intrinsic implications of the two distinct procedures for other settings. Detailed empirical studies are described next to evaluate and compare their accuracy under a wide variety of model configurations.

**Table 1:** Computed sample size, estimated power, and difference for the exact and approximate procedures with {β_*I*_, β_*S*_} = {4.1, 0.15}, {β_*I*0_, β_*S*0_} = {4.198, 0.143}, σ^2^ = 0.095, μ_*X*_ = 24.2, ${\sigma}_X^2$ = 6, and Type I error α = 0.05

Nominal power	Exact approach		Approximate method		Difference	
	*N*	Estimated power	*N*	Estimated power	*N*	Power
0.80	173	0.8001	183	0.8236	10	0.0235
0.90	227	0.9010	239	0.9161	12	0.0151

### Numerical comparisons

In view of the potential discrepancy between the exact and approximate procedures, numerical investigations of power and sample size calculations were conducted under a wide range of model configurations in two studies. The first assessment focuses on the situations with normal predictor variables, while the second study concerns the robustness of the two methods under several prominent situations of non-normal predictors.

#### Normal predictors

For ease of comparison, the model settings in Colosimo et al. [22] are considered and expanded to reveal the distinct behavior of the contending procedures. Specifically, the null and alternative hypotheses are

$$ {\mathrm{H}}_0:\kern1em \left[\begin{array}{c}{\upbeta}_I\\ {}{\upbeta}_S\end{array}\right]=\left[\begin{array}{c}0\\ {}1\end{array}\right]\kern0.5em \mathrm{versus}\ {\mathrm{H}}_1:\kern0.5em \left[\begin{array}{c}{\upbeta}_I\\ {}{\upbeta}_S\end{array}\right]\ne \left[\begin{array}{c}0\\ {}1\end{array}\right], $$

where {β_*I*_, β_*S*_} = {*d*, 1 + *d*} and {β_*ID*_, β_*SD*_} = {*d*, *d*} with *d* = 0.3, 0.4, and 0.5. Note that these coefficient settings are equivalent to those with {β_*I*_, β_*S*_} = {β_*I*0_ + *d*, β_*S*0_ + *d*} because they lead to the same differences {β_*ID*_, β_*SD*_} = {*d*, *d*} and the resulting power functions remain identical. The error component is fixed as σ^2^ = 1 and the predictors *X* are assumed to have normal distributions with mean μ_X_ = {0, 0.5, 1} and variance ${\upsigma}_X^2$ = {0.5, 1, 2}. Overall these considerations result in a total of 27 different combined settings. These combinations of model configurations were chosen to represent the possible characteristics that are likely to be encountered in actual applications and also to maintain a reasonable range for the magnitudes of sample size without making unrealistic assessments.

Throughout this empirical investigation, the significance level and nominal power are fixed as α = 0.05 and 1 – β = 0.90, respectively. With the prescribed specifications, the required sample sizes are computed for the exact procedure with the power function Ψ_*J*_. The computed sample sizes of the nine combined predictor mean and variance patterns are summarized in Table 2, Table S1 and Table S2 for the coefficient difference *d* = 0.3, 0.4, and 0.5, respectively. As suggested by a referee, Tables S1 and S2 are presented in Additional files 3 and 4, respectively. In order to evaluate the accuracy of power calculations, the estimated power of the exact and approximate procedures are also presented. Note that the attained values of the exact approach are marginally larger than the nominal level 0.90. In contrast, the estimated powers of the approximation of Colosimo et al. [22] are all less than 0.90 and the difference is quite substantial in some cases. Then, Monte Carlo simulation studies of 10,000 iterations were performed to compute the simulated power for the designated sample sizes and parameter configurations. For each replicate, *N* predictor values were generated from the designated normal distribution *N*(μ_X_, ${\upsigma}_X^2$). The resulting values of normal predictor, intercept and slope coefficients {β_*I*_, β_*S*_}, and error variance σ^2^, in turn, determine the configurations for producing *N* normal outcomes of the simple linear regression model defined in Eq. 1. Next, the test statistic *F*_*J*_ was computed and the simulated power was the proportion of the 10,000 replicates whose test statistics *F*_*J*_ exceed the corresponding critical value *F*_2, ν, 0.05_. The adequacy of the two sample size procedures is determined by the error between the estimate power and the simulated power. The simulated power and error are also summarized in Table 2, Table S1 and Table S2 for all twenty-seven design schemes.

**Table 2:** Computed sample size, estimated power, and simulated power for Normal predictors with {β_*I*_, β_*S*_} = {0.3, 1.3}, {β_*I*0_, β_*S*0_} = {0, 1}, σ^2^ = 1, Type I error α = 0.05, and nominal power 1 – β = 0.90

μ_*X*_	${\sigma}_X^2$	*N*	Simulated power	Exact approach		Approximate method	
				Simulated power	Error	Simulated power	Error
0	0.5	99	0.9049	0.9025	−0.0024	0.7524	−0.1525
	1	76	0.8997	0.9030	0.0033	0.6257	−0.2740
	2	53	0.9058	0.9050	−0.0008	0.4602	−0.4456
0.5	0.5	56	0.9029	0.9055	0.0026	0.8430	−0.0599
	1	48	0.8993	0.9024	0.0031	0.7756	−0.1237
	2	38	0.8997	0.9006	0.0009	0.6604	−0.2393
1	0.5	35	0.9015	0.9013	−0.0002	0.8682	−0.0333
	1	33	0.9075	0.9089	0.0014	0.8445	−0.0630
	2	28	0.8993	0.9016	0.0023	0.7689	−0.1304

It can be seen from these results that the discrepancy between the estimated power and the simulated power is considerably small for the proposed exact technique for all model configurations considered here. Specifically, the resulting errors of the 27 designs are all within the small range of − 0.0087 to 0.0056. On the other hand, the estimated powers of the approximate method are constantly smaller than the simulated powers. The outcomes show a clear pattern that absolute error decreases with coefficient difference *d* and predictor mean μ_*X*_, and increases with predictor variance ${\upsigma}_X^2$, when all other configurations are held constant. Notably, the associated absolute errors can be as large as 0.4456, 0.4295, and 0.4183 when μ_*X*_ = 0 and ${\upsigma}_X^2$ = 2 for *d* = 0.3, 0.4, and 0.5 in Table 2, Table S1, Table S2, respectively. It should be noted that most of the sample sizes reported in the empirical examination of Colosimo et al. [22] (Table 1) are rather large and impractical. This may explain why the performance of the approximate formula was acceptable in their study. In fact, some of their cases with smaller sample sizes also showed the same phenomenon that the simple method leads to an underestimate of power level and an overestimated sample size required to achieve the nominal power. Essentially, the simplicity of the approximate formula does come with a huge price in terms of inaccurate power and sample size calculations.

#### Non-normal predictors

To address the sensitivity issues of the two techniques, power and sample size calculations were also conducted for the regression models with non-normal predictors. For illustration, the model settings in Table 2 with {β_*ID*_, β_*SD*_} = {0.3, 0.3} are modified by assuming the predictors have four different sets of distributions: Exponential(1), Gamma(2, 1), Laplace(1), and Uniform(0, 1). For ease of comparison, the designated distributions were linearly transformed to have mean μ_*X*_ and variance ${\upsigma}_X^2$ as reported in the previous study. Hence, the computed sample sizes associated with the exact procedure and estimated powers of the two methods remain identical for the four non-normal distributions. The simulated powers were obtained with the Monte Carlo simulation studies of 10,000 iterations under the selected model configurations and non-normal predictor distributions. Similar to the numerical assessments in the preceding study, the computed sample sizes, simulated powers, estimated powers, and associated errors of the two competing procedures are presented in Tables S3-S6 of Additional files 5, 6, 7, 8 for the four types of non-normal predictors, respectively.

Regarding the robustness properties of the two procedures, the results in suggest that the performance of the exact approach is slightly affected by the non-normal covariate settings. The high skewness and kurtosis of the Exponential distribution apparently has a more prominent impact on the normal-based power function than the other three cases of Gamma, Laplace, and Uniform distributions. Note that the approximate method only depends on the mean values of the predictors and is presumably less sensitive to the variation of predictor distributions. However, the accuracy marginally improved in some cases, but generally maintains almost the same performance as in the normal setting presented in Table 2. In short, the sensitivity and robustness of the suggested exact technique depends on the level of how badly predictor distributions depart from normality structure. On the other hand, the performance assessments show that the exact procedure still give acceptable results even in the situations with non-normal predictors considered here. More importantly, these empirical evidences reveal that the exact approach is relatively more reliable and accurate than the approximate method to be recommended as a trustworthy technique for power and sample calculations.

## Discussion

In practice, a research study requires adequate statistical power and sufficient sample size to detect scientifically credible effects. Although multiple linear regression is a well-recognized statistical tool, the corresponding power and sample size problem for model validation has not been adequately examined in the literature. To enhance the usefulness of the joint test of intercept and slope coefficients in linear regression analysis, this article presents theoretical discussions and computational algorithms for power and sample size calculations under the random modeling framework. The stochastic nature of predictor variables is taken into account by assuming that they have an independent and identical normal distribution. In contrast, the existing method of Colosimo et al. [22] adopted a direct replacement of mean values for the predictor variables. Consequently, the proposed exact approach has the prominent advantage of accommodating the complete distributional features of normal predictors whereas the simple approximation of Colosimo et al. [22] only includes the mean parameters of the predictor variables.

## Conclusions

The presented analytic derivations and empirical results indicate that the approximate formula of Colosimo et al. [22] generally does not give accurate power and sample size calculations. According to the overall accuracy and robustness, the exact approach clearly outperforms the approximate methods as a useful tool in planning validation study. Although the numerical illustration only involves a predictor variable, it embodies the underlying principle and critical feature of linear regression that can be useful in conducting similar evaluations for the more general framework of multiple linear regression.

## Additional files

Additional file 1: SAS/IML program for computing the power for the joint test of intercept and slope coefficients. (PDF 60 kb)

Additional file 2: SAS/IML program for computing the sample size for the joint test of intercept and slope coefficients. (PDF 60 kb)

Additional file 3: **Table S1.** Computed sample size, estimated power, and simulated power for Normal predictors with {β_*I*_, β_*S*_} = {0.4, 1.4}, {β_*I*0_, β_*S*0_} = {0, 1}, σ^2^ = 1, Type I error α = 0.05, and nominal power 1 – β = 0.90. (PDF 95 kb)

Additional file 4: **Table S2.** Computed sample size, estimated power, and simulated power for Normal predictors with {β_*I*_, β_*S*_} = {0.5, 1.5}, {β_*I*0_, β_*S*0_} = {0, 1}, σ^2^ = 1, Type I error α = 0.05, and nominal power 1 – β = 0.90. (PDF 96 kb)

Additional file 5: **Table S3.** Computed sample size, estimated power, and simulated power for transformed Exponential predictors with {β_*I*_, β_*S*_} = {0.3, 1.3}, {β_*I*0_, β_*S*0_} = {0, 1}, σ^2^ = 1, Type I error α = 0.05, and nominal power 1 – β = 0.90. (PDF 95 kb)

Additional file 6: **Table S4.** Computed sample size, estimated power, and simulated power for transformed Gamma predictors with {β_*I*_, β_*S*_} = {0.3, 1.3}, {β_*I*0_, β_*S*0_} = {0, 1}, σ^2^ = 1, Type I error α = 0.05, and nominal power 1 – β = 0.90. (PDF 95 kb)

Additional file 7: **Table S5.** Computed sample size, estimated power, and simulated power for transformed Laplace predictors with {β_*I*_, β_*S*_} = {0.3, 1.3}, {β_*I*0_, β_*S*0_} = {0, 1}, σ^2^ = 1, Type I error α = 0.05, and nominal power 1 – β = 0.90. (PDF 95 kb)

Additional file 8: **Table S6.** Computed sample size, estimated power, and simulated power for transformed Uniform predictors with {β_*I*_, β_*S*_} = {0.3, 1.3}, {β_*I*0_, β_*S*0_} = {0, 1}, σ^2^ = 1, Type I error α = 0.05, and nominal power 1 – β = 0.90. (PDF 96 kb)

## Abbreviations

ANCOVA: Analysis of covariance
ANOVA: Analysis of variance

## References

[1] Cohen J, Cohen P, West SG, Aiken LS (2003). Applied multiple regression/correlation analysis for the behavioral sciences.

[2] Kutner MH, Nachtsheim CJ, Neter J, Li W (2005). Applied linear statistical models.

[3] Montgomery DC, Peck EA, Vining GG (2012). Introduction to linear regression analysis.

[4] Snee RD (1977). Validation of regression models: methods and examples. Technometrics.

[5] Maddahi J, Crues J, Berman DS (1987). Noninvasive quantification of left ventricular myocardial mass by gated proton nuclear magnetic resonance imaging. J Am Coll Cardiol.

[6] Rose BI, McCallum WD (1987). A simplified method for estimating fetal weight using ultrasound measurements. Obstet Gynecol.

[7] Cohen J (1988). Statistical power analysis for the behavioral sciences.

[8] Kraemer HC, Blasey C (2015). How many subjects?: Statistical power analysis in research.

[9] Murphy KR, Myors B, Wolach A (2014). Statistical power analysis: a simple and general model for traditional and modern hypothesis tests.

[10] Ryan TP (2013). Sample size determination and power.

[11] Gatsonis C, Sampson AR (1989). Multiple correlation: exact power and sample size calculations. Psychol Bull.

[12] Mendoza JL, Stafford KL (2001). Confidence interval, power calculation, and sample size estimation for the squared multiple correlation coefficient under the fixed and random regression models: a computer program and useful standard tables. Educ Psychol Meas.

[13] Sampson AR (1974). A tale of two regressions. J Am Stat Assoc.

[14] Shieh G (2006). Exact interval estimation, power calculation and sample size determination in normal correlation analysis. Psychometrika.

[15] Shieh G (2007). A unified approach to power calculation and sample size determination for random regression models. Psychometrika.

[16] Shieh G (2009). Exact analysis of squared cross-validity coefficient in predictive regression models. Multivar Behav Res.

[17] Kelley K (2008). Sample size planning for the squared multiple correlation coefficient: accuracy in parameter estimation via narrow confidence intervals. Multivar Behav Res.

[18] Krishnamoorthy K, Xia Y (2008). Sample size calculation for estimating or testing a nonzero squared multiple correlation coefficient. Multivar Behav Res.

[19] Shieh G (2013). Sample size requirements for interval estimation of the strength of association effect sizes in multiple regression analysis. Psicothema.

[20] Shieh G (2017). Power and sample size calculations for contrast analysis in ANCOVA. Multivar Behav Res.

[21] Tang Y (2018). Exact and approximate power and sample size calculations for analysis of covariance in randomized clinical trials with or without stratification. Stat Biopharm Res.

[22] Colosimo EA, Cruz FR, Miranda JLO (2007). Sample size calculation for method validation using linear regression. J Stat Comput Simul.

[23] Binkley JK, Abbot PC (1987). The fixed *X* assumption in econometrics: can the textbooks be trusted?. Am Stat.

[24] Cramer EM, Appelbaum MI (1978). The validity of polynomial regression in the random regression model. Rev Educ Res.

[25] Shaffer JP (1991). The Gauss-Markov theorem and random regressors. Am Stat.

[26] Rencher AC, Schaalje GB (2007). Linear models in statistics.

[27] Anderson NG, Jolley IJ, Wells JE (2007). Sonographic estimation of fetal weight: comparison of bias, precision and consistency using 12 different formulae. Ultrasound Obstet Gynecol.

