# Supplementary material for: Sample size calculations for model validation in linear regression analysis
Source: BMC Med Res Methodol. 2019 Mar 12;19:54. doi: 10.1186/s12874-019-0697-9 (PMC6416874; doi:10.1186/s12874-019-0697-9)
Supplement: Supplementary file 2 — SAS/IML program for computing the sample size for the joint test of intercept and slope coefficients. (PDF 60 kb) [file 12874_2019_697_MOESM2_ESM.pdf]

Supplement program B

SAS/IML program for computing the sample size for the joint test of intercept and slope coefficients

```
PROC IML;
*USER SPECIFICATION PORTION;
*ALPHA;ALPHA=0.05;
*NOMINAL POWER;POWER=0.8;
*INTERCEPT;BETAI=4.1;
*SLOPE: Px1;BETAS={0.15};
*NULL INTERCEPT;BETAI0=4.198;
*NULL SLOPE: Px1;BETAS0={0.143};
*ERROR VARIANCE;VAR=0.095;
*PREDICTOR MEAN: Px1;MUX={24.2};
*PREDICTOR VARIANCE: PxP;VARX={6};
*END OF USER SPECIFICATION PORTION;

BETAID=BETAI-BETAI0;BETASD=BETAS-BETAS0;P=NROW(BETAS);
SIGMA=SQRT(VAR);SIGMAX=SQRT(VARX);
D=BETASD`*VARX*BETASD;A=BETAID+BETASD`*MUX;DELTAJA=(A##2+D)/VA
R;
PRINT ALPHA P POWER;
PRINT BETAI BETAI0 BETAID BETAS BETAS0 BETASD SIGMA VAR;
PRINT MUX SIGMAX VARX DELTAJA[FORMAT=8.4];
NUMINT=200;L=NUMINT+1;DD=1E-6;
COEVEC=({1}||REPEAT({4 2},1,NUMINT/2-1)||{4 1})`;
START POWERISF;
DF=N-P-1;FCRIT=FINV(1-ALPHA,P+1,DF);DFK=N-1;
CL=DD;CU=QUANTILE('CHISQ',1-DD,DFK);
INTC=(CU-CL)/NUMINT;CVEC=CL+(INTC#(0:NUMINT))`;
WCPDF=(INTC/3)#COEVEC#PDF('CHISQ',CVEC,DFK);
ZU=5;ZL=-5;INTZ=(ZU-ZL)/NUMINT;ZVEC=ZL+(INTZ#(0:NUMINT))`;
WZPDF=(INTZ/3)#COEVEC#PDF('NORMAL',ZVEC);
B=SQRT(D/N);
QUAN=J(L,1,0);
DO I=1 TO L;
Z=ZVEC[I,1];
DELTA2VEC=(N#(A+B#Z)##2+D#CVEC)/VAR;
QUAN[I,1]=WCPDF`*SDF('F',FCRIT,2,DF,DELTA2VEC);
```

```
END;
POWERIS=WZPDF`*QUAN;
FINISH;
N=10;LOOP=0;
DO UNTIL (POWERIS>POWER | LOOP>1000);
N=N+1;LOOP=LOOP+1;RUN POWERISF;END;
PRINT N POWERIS[FORMAT=8.4];
QUIT;
```
