# Supplementary material for: Sample size calculations for model validation in linear regression analysis
Source: BMC Med Res Methodol. 2019 Mar 12;19:54. doi: 10.1186/s12874-019-0697-9 (PMC6416874; doi:10.1186/s12874-019-0697-9)
Supplement: Supplementary file 3 — Table S1. Computed sample size, estimated power, and simulated power for Normal predictors with {βI, βS} = {0.4, 1.4}, {βI0, βS0} = {0, 1}, σ2 = 1, Type I error α = 0.05, and nominal power 1 – β = 0.90. (PDF 95 kb) [file 12874_2019_697_MOESM3_ESM.pdf]

Table S1 Computed sample size, estimated power, and simulated power for Normal predictors with $\{\beta_I, \beta_S\} = \{0.4, 1.4\}$, $\{\beta_{I0}, \beta_{S0}\} = \{0, 1\}$, $\sigma^2 = 1$, Type I error $\alpha = 0.05$, and nominal power $1 - \beta = 0.90$

				Exact approach		Approximate method	
μ_X	σ_X^2	N	Simulated power	Estimated power	Error	Estimated power	Error
0	0.5	58	0.9012	0.9044	0.0032	0.7604	–0.1408
	1	45	0.9057	0.9037	–0.0020	0.6358	–0.2699
	2	32	0.9014	0.9043	0.0029	0.4719	–0.4295
0.5	0.5	33	0.9031	0.9033	0.0002	0.8445	–0.0586
	1	29	0.9033	0.9035	0.0002	0.7861	–0.1172
	2	24	0.9171	0.9084	–0.0087	0.6890	–0.2281
1	0.5	22	0.9125	0.9122	–0.0003	0.8848	–0.0277
	1	20	0.9034	0.9035	0.0001	0.8449	–0.0585
	2	18	0.9048	0.9082	0.0034	0.7938	–0.1110
