# Supplementary material for: Sample size calculations for model validation in linear regression analysis
Source: BMC Med Res Methodol. 2019 Mar 12;19:54. doi: 10.1186/s12874-019-0697-9 (PMC6416874; doi:10.1186/s12874-019-0697-9)
Supplement: Supplementary file 4 — Table S2. Computed sample size, estimated power, and simulated power for Normal predictors with {βI, βS} = {0.5, 1.5}, {βI0, βS0} = {0, 1}, σ2 = 1, Type I error α = 0.05, and nominal power 1 – β = 0.90. (PDF 96 kb) [file 12874_2019_697_MOESM4_ESM.pdf]

Table S2 Computed sample size, estimated power, and simulated power for Normal predictors with $\{\beta_I, \beta_S\}$ = {0.5, 1.5}, $\{\beta_{I0}, \beta_{S0}\}$ = {0, 1}, $\sigma^2 = 1$, Type I error $\alpha = 0.05$, and nominal power $1 - \beta = 0.90$

				Exact approach		Approximate method	
μ_X	σ_X^2	N	Simulated power	Estimated power	Error	Estimated power	Error
0	0.5	39	0.9064	0.9066	0.0002	0.7698	–0.1366
	1	31	0.9048	0.9083	0.0035	0.6542	–0.2506
	2	22	0.8988	0.9001	0.0013	0.4805	–0.4183
0.5	0.5	23	0.9049	0.9100	0.0051	0.8583	–0.0466
	1	20	0.9026	0.9016	–0.0010	0.7931	–0.1095
	2	17	0.9101	0.9076	–0.0025	0.7053	–0.2048
1	0.5	15	0.8964	0.9020	0.0056	0.8768	–0.0196
	1	15	0.9210	0.9215	0.0005	0.8768	–0.0442
	2	13	0.9066	0.9062	–0.0004	0.8059	–0.1007
