# Supplementary material for: Sample size calculations for model validation in linear regression analysis
Source: BMC Med Res Methodol. 2019 Mar 12;19:54. doi: 10.1186/s12874-019-0697-9 (PMC6416874; doi:10.1186/s12874-019-0697-9)
Supplement: Supplementary file 7 — Table S5. Computed sample size, estimated power, and simulated power for transformed Laplace predictors with {βI, βS} = {0.3, 1.3}, {βI0, βS0} = {0, 1}, σ2 = 1, Type I error α = 0.05, and nominal power 1 – β = 0.90. (PDF 95 kb) [file 12874_2019_697_MOESM7_ESM.pdf]

Table S5 Computed sample size, estimated power, and simulated power for transformed Laplace predictors with $\{\beta_I, \beta_S\} = \{0.3, 1.3\}$, $\{\beta_{I0}, \beta_{S0}\} = \{0, 1\}$, $\sigma^2 = 1$, Type I error $\alpha = 0.05$, and nominal power $1 - \beta = 0.90$

				Exact approach		Approximate method	
μ_X	σ_X^2	N	Simulated power	Estimated power	Error	Estimated power	Error
0	0.5	99	0.8994	0.9025	0.0031	0.7524	–0.1470
	1	76	0.8948	0.9030	0.0082	0.6257	–0.2691
	2	53	0.8897	0.9050	0.0153	0.4602	–0.4295
0.5	0.5	56	0.9060	0.9055	–0.0005	0.8430	–0.0630
	1	48	0.8975	0.9024	0.0049	0.7756	–0.1219
	2	38	0.8903	0.9006	0.0103	0.6604	–0.2299
1	0.5	35	0.8965	0.9013	0.0048	0.8682	–0.0283
	1	33	0.9058	0.9089	0.0031	0.8445	–0.0613
	2	28	0.9011	0.9016	0.0005	0.7689	–0.1322
