# Supplementary material for: Sample size calculations for model validation in linear regression analysis
Source: BMC Med Res Methodol. 2019 Mar 12;19:54. doi: 10.1186/s12874-019-0697-9 (PMC6416874; doi:10.1186/s12874-019-0697-9)
Supplement: Supplementary file 8 — Table S6. Computed sample size, estimated power, and simulated power for transformed Uniform predictors with {βI, βS} = {0.3, 1.3}, {βI0, βS0} = {0, 1}, σ2 = 1, Type I error α = 0.05, and nominal power 1 – β = 0.90. (PDF 96 kb) [file 12874_2019_697_MOESM8_ESM.pdf]

Table S6 Computed sample size, estimated power, and simulated power for transformed Uniform predictors with $\{\beta_I, \beta_S\} = \{0.3, 1.3\}$, $\{\beta_{I0}, \beta_{S0}\} = \{0, 1\}$, $\sigma^2 = 1$, Type I error $\alpha = 0.05$, and nominal power $1 - \beta = 0.90$

				Exact approach		Approximate method	
μ_X	σ_X^2	N	Simulated power	Estimated power	Error	Estimated power	Error
0	0.5	99	0.8971	0.9025	0.0054	0.7524	–0.1447
	1	76	0.9101	0.9030	–0.0071	0.6257	–0.2844
	2	53	0.9076	0.9050	–0.0026	0.4602	–0.4474
0.5	0.5	56	0.9078	0.9055	–0.0023	0.8430	–0.0648
	1	48	0.9000	0.9024	0.0024	0.7756	–0.1244
	2	38	0.9036	0.9006	–0.0030	0.6604	–0.2432
1	0.5	35	0.8990	0.9013	0.0023	0.8682	–0.0308
	1	33	0.9124	0.9089	–0.0035	0.8445	–0.0679
	2	28	0.8994	0.9016	0.0022	0.7689	–0.1305
